# Determining the validity and reliability of the Chinese version of the Kidney Disease Quality of Life Questionnaire (KDQOL-36^™^)

**DOI:** 10.1186/1471-2369-15-115

**Published:** 2014-07-11

**Authors:** Xingjuan Tao, Susan Ka Yee Chow, Frances Kam Yuet Wong

**Affiliations:** 1 School of Nursing, The Hong Kong Polytechnic University, Hunghom, Kowloon, Hong Kong

## Abstract

**Background:**

Health-related quality of life is a crucial outcome for the chronic kidney disease population, the Kidney Disease Quality of Life (KDQOL) questionnaire is commonly used as an integral part of clinical evaluations. The abbreviated version of the KDQOL-36™ has been translated into Mandarin Chinese, but has not been tested for use in the Chinese patients. The aim of the study was to evaluate the validity and reliability of the KDQOL-36™ with a sample of Chinese patients.

**Methods:**

The Mandarin Chinese version of the KDQOL-36™ has been translated by Amgen, Inc. and the MAPI Institution following the instrument translation specifications provided by the RAND health. The translated instrument was further reviewed by a Chinese expert panel for content validity and translational equivalence. The KDQOL-36™ along with Beck depression Inventory-II were administrated to 103 chronic renal disease patients recruited through convenience sampling procedure from the renal wards and an outpatient dialysis clinic. The convergent validity was determined through investigating the correlational evidence of the KDQOL-36™ with the Beck depression Inventory-II and the overall health rating. Known-group validity was supported by the evidence that the instrument could differentiate subgroups of patients. The internal consistency was estimated using Cronbach’s α and test-retest reliability was examined using an intraclass correlation coefficient.

**Results:**

For the convergent validity, there were positive correlations between the overall health rating and most of the KDQOL-36™ subscales, and the Beck depression inventory score was inversely correlated with the mental component summary score and disease-specific scores of the scale. Significant correlations were noted between disease-targeted and generic dimensions of the KDQOL-36™. The results of the known-group comparisons indicated females, the unemployed, and patients had a longer dialysis history reported a worse quality of life. With regard to the reliability, the Cronbach’s alpha ranged from 0.69 to 0.78, and the intraclass correlation test-retest was higher than 0.70.

**Conclusions:**

The Mandarin Chinese version of the KDQOL-36™ is a brief, valid, and reliable instrument for use in examining the quality of life of chronic kidney disease patients in China.

## Background

Chronic kidney disease (CKD) has become a major global health issue, affecting around 10-13% of the adult population in countries such as the US [1], Taiwan [2], and China [3]. Owing to the progressive nature of CKD, patients with CKD are at a high risk of gradually progressing to end stage renal disease (ESRD). The estimate in current available reports is that approximately 1.9 million ESRD patients around the world are receiving renal replacement therapy (RRT); the figure does not capture the proportion of ESRD patients without access to RRT treatment [4]. Life-long dialysis and kidney transplantation are treatment choices for patients with ESRD to sustain their lives. These patients suffer from the signs and symptoms of relapse, experience fear and anxiety, and face financial burdens, causing an impaired quality of life [5]. In mainland China, the number of CKD patients was estimated to be around 119.5 million based on a national survey in 2010 [3]. Data from the Chinese Renal Data System revealed that there were about 270,000 patients undergoing haemodialysis (HD), while only 30,000 were received peritoneal dialysis (PD) treatment at the end of 2012 [6], suggesting that HD is the major treatment modality in China, accounting for approximately 90% of the total ESRD patients.

Chronic kidney disease is a progressive disease. Patients with CKD experienced impaired health-related quality of life (HRQOL) [7-9], as both the renal disease itself and its treatment have long-term impacts on lifestyle. Accumulating studies have generated evidence to support the relationships between impaired HRQOL and clinical outcomes, such as increased hospitalization and mortality, in CKD population [10-12]. Attention has recently focused on effects to improve the HRQOL of persons with CKD. HRQOL is increasingly being chosen over laboratory data as the primary outcome measure in clinical research [13] when examining the effects of treatment, because an improvement in HRQOL would bring greater fulfillment to the lives of patients [10]. The routine use of HRQOL measures creates an opportunity for health care providers to incorporate the experiences of patients when developing interventions that best suit their needs given the patients’ adverse life circumstances [14], and to support patient-centered treatment decisions [15].

The Kidney Disease Quality of Life (KDQOL™) questionnaire combines the generic SF-36 instrument and disease-specific components for assessing the HRQOL of CKD patients. The original questionnaire consists of 134 items, and takes about 30 minutes to complete [16]. The authors further developed a short version – the KDQOL-SF™ version 1.3 – which includes 43 items focusing on kidney disease and SF-36 [17]. The KDQOL-SF™ has been translated into many languages including Chinese. The latest abbreviated version – KDQOL-36™ – is even briefer making it more likely that patients will respond to the questionnaire [18]. The brief version has also been found to be suitable for use in routine evaluations of quality of care in busy practice settings [19] and has been used extensively to evaluate CKD patients in different stages of the disease, including patients who are receiving dialysis treatment [20].

The Mandarin Chinese version of the scale was translated by Amgen, Inc. and the MAPI Institute and can be downloaded from the website of the RAND Corporation [21] for non-commercial use. The translation process followed the specific guidelines stipulated by the RAND health [22]. Forward and back forward translations were adopted to ensure the equivalence between the original English version of the KDQOL-36™ and the translated Mandarin Chinese version. The psychometric properties of the translated version of the scale have not been evaluated or reviewed by RAND. Chinese comprise 19% of the world’s total population. The number of patients in China with CKD is estimated to be about 119.5 million [3]. A valid Chinese version of the KDQOL-36™ will enable researchers to examine the quality of life of CKD patients within the country and allow for cross-country comparisons to be made. To address this need, the aim of the study was to determine the validity and reliability of the KDQOL-36™ with a Chinese population.

## Methods

The study was approved by the Human Subjects Ethical Sub-committee of the Hong Kong Polytechnic University and by the hospital in which the study would be conducted. The participants were informed that their participation was voluntary and that they could withdraw at any stage.

### Sampling and data collection method

One hundred and three patients with mild-to-severe CKD were recruited from the renal wards and outpatient dialysis clinics of a tertiary hospital in mainland China through convenience sampling. This study included both CKD patients who had commenced dialysis treatment and those who had not. The criteria for inclusion were patients who had been diagnosed with CKD, who were above the age of 18, and who were able to respond to the questionnaire. The criteria for exclusion were patients who had been diagnosed with a mental illness. Hobart et al. suggested that the minimum sample size required for testing the validity and reliability of an instrument is 80 and 20 subjects, respectively [23]. A sample of 50 subjects or more is required to determine the internal consistency of a five-point scale [24]. Based on the two recommendations, the sample size of the study was determined to be no less than 80. The number of subjects needed to determine the test-retest reliability of the KDQOL-36™ was estimated using the intraclass correlation coefficient (ICC) value. To achieve a specificity of 0.95 and a power of 0.5, and assuming an ICC value of 0.8 for the instrument with two occasional observations, a sample size of 22 would suffice to allow for observations of ICC values of 0.5 or greater [25].

### Questionnaires used in the study

The participants completed a self-administered questionnaire including the KDQOL-36™, Beck Depression Inventory (BDI-II) and a demographic questionnaire. Most of the questionnaires were self-administered, with nurses providing assistance to those patients who were unable to complete the questionnaires on their own. The disease-specific core of KDQOL-36™ has 24 items comprising three scales: Symptoms and Problems (12 items), Burden of Kidney Disease (4 items), and Effects of Kidney Disease (8 items). The generic core is the 12-item Short Form Health Survey (SF-12) [26]. The raw scores are transformed linearly to a range of 0 to 100, with higher scores indicating better HRQOL [17]. A specific item related to dialysis access was left blank by patients who were not receiving dialysis. The blank item was not used to calculate the score, according to the KDQOL 1.3 Manual for Use and Scoring [17]. The results of the SF-12 instrument were summarized into the Physical Component Summary (PCS) score and the Mental Component Summary (MCS) score. The BDI-II was used to assess the presence and intensity of depressive symptoms in clinically depressed or non-depressed patients. Each item measured via a four-point scale (0–3) corresponding to a symptom of depression is summed up to give a single score for the BDI-II [27]. The total score ranges from 0 to 63, with higher scores indicating severe depression. The construct validity of the original English version was supported by the criterion-related validation and the convergent validation approach [28]. The Mandarin Chinese version of the BDI-II has good internal consistency with a Cronbach’s alpha coefficient of 0.94. The construct validity was supported by the concurrent validity and exploratory factor analysis approach [29]. A questionnaire on the demographic and clinical information of the patients was developed by the authors. The items included questions on age, gender, marital status, education level, primary causes of CKD, complications, and dialysis vintage if the patients were receiving dialysis treatment.

### Validity and reliability testing of the Chinese KDQOL-36™

#### Validity

Content validity is about whether a scale adequately samples all possible and relevant questions that exist in terms of its content [30]. An expert panel that included two academic researchers, two clinical renal nurses, and a renal physician was formed to examine the translation equivalence and contents of the instrument. Content validity was assessed using a four-point Likert scale (1 = ‘not relevant’, 2 = ‘somewhat relevant’, 3 = ‘quite relevant’, 4 = ‘very relevant’) based on the cultural relevance of each item in measuring HRQOL among CKD patients. Both the item-level content validity index (CVI) and scale-level CVI were calculated. The item-level CVI (I-CVI) was computed as the number of experts giving a rating of either 3 or 4, divided by the number of experts [31]. The scale-level CVI (S-CVI) was generated as the percentage of items on the questionnaire that obtained a rating of 3 or 4 from all of the reviewers [32].

Convergent validity involves investigating the correlational evidence of a measurement under development using another scale [30]. The overall health rating and BDI score were selected to test their correlations with KDQOL™-36 subscale scores [33]. Previous studies have shown that depressive symptoms among the CKD population are strongly associated with poor health-related quality of life in multiple domains [34,35]. We hypothesized that patients with lower subscale scores on the KDQOL-36™ would report higher levels of depressive symptoms, as represented by a high BDI-II score. In addition, the correlation between the overall health rating and the KDQOL-36™ was examined. The overall health rating was examined through the first item of the KDQOL-36™, a global measure of an individual’s HRQOL [36]. With regard to the impact of the overall health rating on the HRQOL, previous studies showed that all subscales of the HRQOL were independently correlated with self-rated health in the CKD patient group [20]. Based on the above established evidence, we hypothesized that each subscale score of the KDQOL-36™ would be positively correlated with the overall health rating.

Known-group comparison is an approach used to detect differences in mean scores between groups that are known to exhibit different traits on a construct of measurement [37]. In this study, we compared differences in the scores of subgroups of patients in terms of demographics and clinical status, such as age, gender, working status, history of hospitalization, disease stages and dialysis duration. Based on previous studies, we assumed that HRQOL scores would be lower among elderly people, females, the poorly educated, the unemployed, and people without government health insurance [38-41]. It was also expected that patients who had been hospitalized during the past six months and patients who had undergone dialysis for a longer duration would report lower HRQOL [38]. The correlations between scores on generic and kidney disease-specific domains were inspected to further establish the convergent validity. The generic and disease-targeted scales were hypothesized to be correlated with each other.

#### Reliability

Reliability pertains to the ability of an instrument to consistently measure an attribute [42]. In our study, evidence of reliability was derived by examining the internal consistency and test-retest reliability. Twenty eight subjects were asked to respond to the same set of questionnaire within ten to fourteen days interval to determine the stability of the scale.

#### Acceptability and response burden

Acceptability was assessed by examining the completion rates and missing data, and identifying the ceiling or floor effects. The response burden was also evaluated using one question: ‘Please evaluate the level of difficulty in responding to this questionnaire’. The available response choices were ‘easy’, ‘moderate’ and ‘difficult’.

#### Data analysis

Data analyses were carried out using the SPSS 20.0 (IBM PASW, USA). All statistical tests were two-tailed and *P <* 0.05 was considered statistically significant. The percentages of patients achieving the highest (100) and lowest scores (0) were calculated to examine the ceiling and floor effects of the questionnaire. Descriptive statistics, such as mean, standard deviation, and percentage, were used to examine the demographic information. The levels of skewness and kurtosis were determined to assess the normality of each variable [43]. Internal consistency reliability was evaluated using the Cronbach’s alpha coefficient calculated separately for each subscale. A coefficient alpha of 0.70 or greater is generally considered to be acceptable [44]. Test-retest reliability was estimated by calculating the ICCs based on the two-way mixed analysis of variance (ICC_3,1_). An ICC of above 0.75 indicates excellent test-retest reliability, 0.40 and 0.75 are considered to be good, while values of below 0.4 indicate weak agreement [45]. For convergent validity, Spearman’s correlation coefficients were used to examine the relationships between subscales of the KDQOL-36™ and the hypothesized measures, as well as correlations between generic and disease-specific domains. A correlation of 0.40 is considered substantial for conceptually related scales [46,47]. Independent *t*-tests and an analysis of variance (ANOVA) for continuous variables were used to evaluate the differences between the hypothesized ‘known’ groups if data were normally distributed.

## Results

### Characteristics of the study subjects

The mean age of the participants in the study was 47.6 years and more than half were male (55.3%). The majority were married (79.6%) and not working (60.2%). With regard to clinical characteristics, chronic glomerulonephritis was the most common cause of CKD (52.4%). For patients who were receiving dialysis, the mean duration of treatment was 45.9 months. For details, refer to Table 1.

**Table 1:** Patients characteristics (n = 103)

**Characteristics**		***n *** **(%)**
Gender	Male	57 (55.3%)
	Female	46 (44.8%)
Age, year (Mean, SD)		47.6 (14.2)
Marital status	Married	82 (79.6%)
	Singled	16 (15.5%)
	Divorced/widowed	5 (4.8%)
Education	Primary school or below	13 (12.6%)
	Secondary school	60 (58.3%)
	College or above	30 (29.1%)
Employment status	Employed	41 (39.8%)
	Unemployed	62 (60.2%)
Health insurance status	Government insurance	73 (70.9%)
	Self-paying	27 (26.2%)
	Other	3 (2.9%)
Primary renal disease	Chronic glomerulonephritis	54 (52.4%)
	Hypertension	12 (11.7%)
	Gouty kidney	10 (9.8%)
	Unknown etiology	8 (7.8%)
	Diabetes	6 (5.8%)
	Systemic lupus erythematosis	4 (3.9%)
	Other	9 (8.7%)
Dialysis	CKD(stage1-4) not commencing dialysis	27 (26.2%)
	Haemodialysis (HD)	49 (47.6%)
	Peritoneal dialysis (PD)	27 (26.2%)
Dialysis duration, month (Mean, SD)		45.9 (41.4)

### Acceptability and descriptive analysis of the scale

The completion rates were high for nearly all of the items in the KDQOL-36™. None of participants selected ‘difficult’ for the subjective burden of answering the questionnaire, and 57.3% of the subjects chose ‘moderate’. The mean scores for each subscale of the KDQOL-36™ ranged from 33.07 to 74.22. Symptoms and problems of kidney disease had the highest mean score (74.22 ± 15.24) while Burden of kidney disease had the lowest mean score (33.07 ± 23.31). Regarding the distribution of the responses to the scales on the KDQOL-36™, ceiling effects were noted in the Symptom/Problem list (2.9%) and in the Burden of Kidney disease (1%) domains. Both the Effect of Kidney Disease (1%) and Burden of Kidney Disease (13.6%) scales demonstrated floor effects. The mean BDI-II score was 17.20 (11.43), ranging from 0 to 50. The descriptive statistics of the KDQOL-36™ are summarized in Table 2.

**Table 2:** **Descriptive statistics of the KDQOL-36™**

**KDQOL-36™ Subscales**	**Mean**	**SD**	**Minimal (Floor%)**	**Maximal (Ceiling%)**
Symptoms/problems list	74.22	15.24	37.50 (1)	100 (2.9)
Effects of kidney disease	54.78	20.10	00.00 (1)	96.88 (1)
Burden of kidney disease	33.07	23.31	00.00 (13.6)	100 (1)
Physical Composite Sore	36.60	7.83	17.05 (1)	54.92 (1)
Mental Composite Score	46.82	9.81	22.17 (1)	65.09 (1)

### Validity estimate

For the content validity, the expert panel commented that the use of ‘bowling’ and ‘playing golf’ as examples of ‘moderate activities’ in the original Mandarin version were not appropriate, as these are forms of exercise that Chinese people do not normally engage in. They were therefore replaced by ‘Walking’ and ‘Tai Chi’ , which the Chinese would be more likely to take up for exercise. The changes were based on the Compendium of Physical Activities [48,49], which lists the levels of energy expended in ‘walking’ and practising ‘Tai Chi’ as similar to those for ‘bowling’ and ‘playing golf’. After the revisions, the panel determined the content validity using a four-point Likert scale. The item-level content validity index (I-CVI) and scale-level CVI (S-CVI) were 1.0.

With regard to convergent validity, significant positive correlations were found between all of the subscale scores and the overall health rating score (*p* < 0.01). Significant negative correlations were found between all of the disease-specific domain scores and the BDI score, from 0.395 to 0.654, whilst the correlation coefficient found between the MCS and BDI scores was higher than the correlation between the PCS and BDI scores. In addition, all kidney disease-specific domains were significantly correlated with two generic component summaries, with coefficients ranging from 0.333 to 0.511. The strongest correlation was found between Burden of Kidney Disease and MCS. No significant correlation was observed between PCS and MCS. For details, refer to Table 3.

**Table 3:** **Correlations between the domains of the KDQOL-36™ with the overall health rating and BDI-II scores**

**KDQOL™-36 subscales**	**Overall health rating score**	**BDI-II score**	**PCS**	**MCS**
Symptoms/problems list	0.462**	-0.506**	0.452**	0.363**
Effects of kidney disease	0.314**	-0.654**	0.329**	0.487**
Burden of kidney disease	0.447**	-0.621**	0.333**	0.511**
PCS	0.499**	-0.395**	1	0.152
MCS	0.377**	-0.483**	0.152	1

Spearman’s rho correlation, **P < 0.01.

With regard to known-group comparisons, females and patients who had been hospitalized during the past 6 months had lower scores on the perception of the Burden of Kidney Disease (*P* < 0.05), whilst patients who had been undergoing dialysis for a longer duration reported lower scores on Symptoms and Problems (*P* < 0.05). With regard to the generic cores of the KDQOL-36™, working patients and patients who had undergone dialysis for a shorter duration had significantly higher PCS scores (*P* < 0.05), while patients without government health insurance had significantly lower MCS scores (*P* < 0.05). Compared to peritoneal dialysis patients and CKD patients who had not commenced dialysis treatment, haemodialysis patients had higher PCS and MCS scores (*P* = 0.036, *P* = 0.006, respectively). For details, refer to Table 4.

**Table 4:** **Subgroup comparisons of the KDQOL-36™**

	**KDQOL-36™ subscales (Mean, SD)**				
	**Symptoms/problems list**	**Burden of kidney disease**	**Effects of kidney disease**	**PCS**	**MCS**
Age					
Age ≤ 45 (*n* = 46)	73.95 (15.19)	36.55 (24.26)	55.97 (20.61)	36.52 (7.45)	46.35 (9.82)
Age = 46-59 (*n* = 36)	73.90 (15.92)	31.60 (34.00)	52.59 (20.28)	37.81 (7.81)	45.66 (10.08)
Age ≥ 60 (*n* = 21)	75.34 (14.82)	27.98 (19.53)	55.93 (19.25)	34.72 (8.62)	49.84 (9.11)
*P* value	0.071	0.341	0.724	0.356	0.275
Gender					
Female (*n* = 46)	72.89 (15.51)	27.04 (21.65)	53.63 (18.09)	36.15 (7.96)	47.26 (9.42)
Male (*n* = 57)	75.29 (15.07)	37.94 (23.65)	55.71 (21.69)	36.97 (7.77)	46.46 (10.18)
*P* value	0.431	0.018^*^	0.604	0.600	0.683
Working status					
Working (*n* = 41)	77.08 (15.36)	55.14 (21.78)	38.72 (24.81)	38.81 (7.95)	45.28 (9.95)
Not working (*n* = 62)	72.32 (14.98)	54.55 (19.08)	29.33 (21.67)	35.14 (7.45)	47.84 (9.66)
*P* value	0.121	0.884	0.045	0.019^*^	0.196
Health insurance					
Government paid (*n* = 73)	74.95 (15.76)	55.25 (19.42)	34.16 (22.29)	36.42 (8.13)	48.33 (9.66)
Self-paid (*n* = 30)	72.44 (13.97)	53.65 (21.97)	30.42 (25.83)	37.06 (7.13)	43.15 (3.92)
*P* value	0.452	0.715	0.462	0.709	0.014*
Presence of Complications					
Yes (*n* = 56)	72.79 (15.42)	52.27 (18.51)	29.91 (20.04)	35.22 (7.16)	45.76 (9.72)
No (*n* = 40)	75.30 (15.44)	57.30 (21.18)	36.72 (27.31)	38.90 (7.45)	47.92 (10.11)
*P* value	0.434	0.219	0.185	0.017^*^	0.294
History of hospitalization					
Yes (*n* = 34)	75.54 (13.93)	25.92 (20.65)	53.56 (18.76)	34.80 (7.60)	46.11 (10.37)
No (*n* = 69)	73.56 (15.90)	36.59 (23.88)	55.38 (20.83)	37.49 (7.84)	47.17 (9.58)
*P* value	0.539	0.028*	0.667	0.101	0.607
Disease stages					
CKD 1–4 (*n* = 27)	77.10 (14.50)	30.79 (24.75)	55.52 (24.11)	36.71 (8.88)	43.08 (10.38)
PD (*n* = 27)	69.75 (14.26)	26.39 (18.62)	48.96 (16.22)	33.46 (5.70)	44.84 (7.65)
HD (*n* = 49)	75.09 (14.50)	38.01 (24.13)	57.58 (19.32)	38.27 (7.84)	49.97 (9.70)
*P* value	0.179	0.096	0.198	0.036*	0.006*
Dialysis duration					
≥60 months (*n* = 24)	68.06 (15.49)	35.16 (21.87)	54.22 (17.63)	33.99 (8.48)	49.22 (9.99)
<60 months (*n* = 52)	75.56 (13.60)	33.29 (23.54)	54.66 (19.24)	37.76 (6.73)	47.66 (9.03)
*P* value	0.036*	0.744	0.926	0.040*	0.501

*P < 0.05. *Note*: There were missing data for “Presence of Complications”, resulting in the number of patients being less than the total sample size. For “Dialysis duration”, there were 27 CKD patients were not receiving dialysis; the data were drawn from 76 dialysis patients.

### Reliability estimate

With regard to internal consistency, the Cronbach’s alpha coefficient for the subscales ranged from 0.69 to 0.78. The subscale for PCS marginally met the recommended criterion for internal reliability. With regard to test-retest reliability, the ICCs ranged from 0.70 to 0.86 for the subscale scores. For details, refer to Table 5.

**Table 5:** **Reliability of the KDQOL-36**^**™ **^**subscales**

**Scales (No. of items)**	**Cronbach’s α (*n*** **= 103)**	**ICC (*n*** **= 28)**
Symptoms/problems list (12)	0.78	0.84
Burden of kidney disease (4)	0.76	0.86
Effects of kidney disease (8)	0.77	0.85
Physical Composite Score (6)	0.69	0.70
Mental Composite Score (6)	0.72	0.81

## Discussion

Mandarin Chinese is spoken by around 850 million people in China, Taiwan, and Southeast Asia, as well as in the US, Canada, New Zealand, Peru, and South Africa [50]. This study is the first validation study of the KDQOL-36™ questionnaire to have been conducted in China. It has demonstrated that the Mandarin Chinese version of the scale is linguistically and culturally relevant to Chinese CKD patients. The ceiling and floor effects were less than 20%, suggesting that the instrument can capture the full range of potential responses in CKD population [51].

Lynn [52] recommended that if five or fewer experts give a rating, the I-CVI must be 1.0. An S-CVI of 0.8 or higher is considered acceptable [32]. Both the I-CVI and S-CVI were 1.0. The results indicated that all items of the Mandarin Chinese version of KDQOL^™^-36 were considered to be appropriate and relevant, giving evidence of the excellent content validity. During the content validity process, ‘walking’ and ‘playing Tai Chi’ were used in place of the problematic examples. Similar amendments were reported for different versions of the KDQOL-36™, such as the Korean [53], Filipino [45], Portuguese [54], and Egyptian [55] versions.

The convergent validity of the KDQOL-36™ was supported by the hypothesis that those patients who experienced better quality of life had a higher overall health rating. The overall health rating reflects the individual’s feelings and provides an estimate of the subjective perception of one’s health status [33]. Substantial correlations were observed between overall health and the subscales for Symptoms and problems, Burden of kidney disease, and PCS (P < 0.01), confirming that the KDQOL-36™ and the overall physical health rating are conceptually related. These results are consistent with studies that have validated versions of the KDQOL™ instrument in other languages, such as the Korean [53], Singaporean [56], Greek [57] and Iranian [51] versions. It was noted that the non-substantial correlation between the MCS score and the overall health score could be related to how an individual perceived his/her overall health. Previous studies have suggested that amongst the general adult population, the overall health rating principally reflects the physical dimension of health [58]. Moreover, there was a non-substantial correlation between the Effects of kidney disease and the overall health rating. As ESRD patients were getting used to the idea that they would need life-long treatment [26], living on dialysis had become their ‘normal way of being’ [59]. To help them to increase their confidence in maintaining their health, some patients even considered dialysis to be a ‘part-time job’ [5]. On the other hand, patients who were receiving dialysis could not avoid fluid or dietary restrictions even if their condition had improved. Therefore, changes in the patients’ perception of their overall health might not have a direct or strong relationship with the Effects of kidney disease, which is consistent with our results.

The convergent validity of the KDQOL-36™ was also supported by the hypothesis that patients with lower subscale scores in the KDQOL-36™ would report a higher BDI-II score. All of the disease-specific domains and the MCS showed substantial inverse correlations with the BDI score. Similar findings were reported in previous studies, showing that depressive symptoms among the CKD population were strongly associated with poor health-related quality of life in multiple domains [34,35]. A relatively low correlation was found between the PCS domain and the BDI score. A possible explanation for this is that our diverse patient groups experienced different stressors. The psychological stress of patients who had not yet commenced dialysis treatment might not have been related to impaired physical functioning, but to obligatory hospital visits or to the fear of becoming dependent on dialysis [60]. The positive correlations coefficients between the kidney disease targeted scales and two main composite summaries from SF-12 demonstrated that the two components (generic core and disease-targeted core) of the KDQOL-36™ are conceptually related. The significant association presented in this study further supports the construct validity of the KDQOL-36™, and is in agreement with the results reported in other validation study [55].

The KDQOL-36™ demonstrated evidence of known-group validity as the scale scores were able to discriminate between subgroups of patients. In our study, females, the unemployed, patients with complications, and patients who had undergone dialysis for a longer duration tended to have worse HRQOL. The results corroborated those of previous studies evaluating the quality of life of CKD patients, where gender, employment status, comorbidities, and dialysis duration were shown to influence HRQOL scores [39,40,58,61,62]. Contrary to our expectations, there were no significant differences in any of the KDQOL-36™ subscale scores among those of different age groups. A possible explanation for this is that more young patients were recruited in our study, with only 20.4% of the participants being older than 60. In addition, the hypothesis that dialysis patients experience a lower HRQOL than those who have not yet commenced dialysis was not supported. This could be due to the fact that the non-dialysis CKD patients recruited in our study were hospitalized, while the dialysis patients were not. Hospitalized patients experience a low HRQOL [63]. Hence, the HRQOL of those hospitalized non-dialysis patients might be lower than that of outpatients who are receiving dialysis, which is consistent with our findings.

For test-retest reliability, an ICC of 0.70-0.86 demonstrated the stability of the scale over time [30]. The Cronbach’s alpha values suggested that the scale is internally reliable. The internal reliability of all of the subscales exceeded 0.7, with the PCS (0.69) approaching the minimum desirable standard. Acceptable levels of internal consistency suggested that all of the items from each subscale of the KDQOL-36™ fit together conceptually and measure the same construct [42].

## Conclusions

The results of our present study support the claim that the Mandarin Chinese version of the KDQOL-36™ is easy to understand and demonstrates good validity and reliability. The evidence supports the view that the questionnaire is culturally appropriate for use in Chinese populations with CKD, and can be adopted by both researchers and health care providers who are interested in understanding and designing interventions to improve the quality of life of patients.

There were some limitations to this study. First, the patients were recruited from a single study site, which may limit the generalizability of the findings. Second, the testing was conducted among a mixed sample of CKD patients with no even distribution according to age. Due to the limited sample size, this could suggest the existence of bias, as the younger patients tended to report a higher HRQOL. The study was conducted using a mixed sample of CKD patients. Although including a wide range of patients allowed variations in quality of life measures, it affected the homogeneity of the sample. A further evaluation of the instrument on a larger Chinese sample is warranted to support our findings.

Validating an instrument is an ongoing process and requires a wide and diverse body of evidence [64]. To accumulate evidence on the construct validity of the questionnaire, future research is needed to examine the internal structure of the KDQOL-36™ by exploratory and confirmatory factor analysis, and to investigate the relationships of the KDQOL-36™ subscales with other external variables using different hypotheses and approaches.

## Competing interests

The authors declare that they have no conflicts of interest to disclose.

## Authors’ contributions

TXJ, SC conceptualized and designed the study. TXJ performed the data collection, data entry and analyzed the data. TXJ and SC wrote the manuscript. All authors read and approved the final manuscript.

## Pre-publication history

The pre-publication history for this paper can be accessed here:

http://www.biomedcentral.com/1471-2369/15/115/prepub
